# Dynamic prediction of repeated events data based on landmarking model: application to colorectal liver metastases data

**DOI:** 10.1186/s12874-019-0677-0

**Published:** 2019-02-14

**Authors:** Isao Yokota, Yutaka Matsuyama

**Affiliations:** 1 Department of Biostatistics, Graduate School of Medicine, Hokkaido University, Kita 15, Nishi 7, Kita-ku, Sapporo, Hokkaido 060-0061 Japan; 2 Department of Biostatistics, School of Public Health, Graduate School of Medicine, The University of Tokyo, Tokyo, Japan

**Keywords:** Dynamic prediction, Landmarking, Pseudo-observations, Repeated events, Terminal event

## Abstract

**Background:**

In some clinical situations, patients experience repeated events of the same type. Among these, cancer recurrences can result in terminal events such as death. Therefore, here we dynamically predicted the risks of repeated and terminal events given longitudinal histories observed before prediction time using dynamic pseudo-observations (DPOs) in a landmarking model.

**Methods:**

The proposed DPOs were calculated using Aalen–Johansen estimator for the event processes described in the multi-state model. Furthermore, in the absence of a terminal event, a more convenient approach without matrix operation was described using the ordering of repeated events. Finally, generalized estimating equations were used to calculate probabilities of repeated and terminal events, which were treated as multinomial outcomes.

**Results:**

Simulation studies were conducted to assess bias and investigate the efficiency of the proposed DPOs in a finite sample. Little bias was detected in DPOs even under relatively heavy censoring, and the method was applied to data from patients with colorectal liver metastases.

**Conclusions:**

The proposed method enabled intuitive interpretations of terminal event settings.

**Electronic supplementary material:**

The online version of this article (10.1186/s12874-019-0677-0) contains supplementary material, which is available to authorized users.

## Background

Events of interest are repeatedly observed during follow-up of some diseases. For example, in colorectal liver metastases following curative surgical resection, the incidence of recurrence is as high as 75% [1]. Surgical re-resection for recurrence is performed when possible, and repeat recurrences are often resected until lesions are unresectable or fatal. The most recent clinical observations of tumors are highly predictive of subsequent recurrence, particularly in patients with multiple tumors or previously resected tumors that may result in recurrence. Risk of recurrence or death can be predicted easily by a conventional approach with time to recurrence-free survival. Prediction of the risk of recurrence and the risk of death separately has more clinical importance because a recurrent case may undergo re-resection, unlike a fatal case, in other words, a recurrent case is still at risk. Furthermore, the prediction of the number of recurrences will be helpful for recognition of its severity. Therefore, personalized predictions of each risk of recurrence and death can be used to communicate his/her prognosis to the patient and decide optimal examination intervals for detecting recurrences.

Dynamic prediction has an intuitive expression associated with the estimated probability of event occurrence within (*t*, *t* + *w*), assuming that the patient is at risk at prediction time *t*. For univariate survival outcomes, this probability can be calculated using the Breslow estimator based on Cox proportional hazards models [2]. However, for repeated events data, dynamic prediction models are required to estimate the probability of numbers of events within (*t*, *t* + *w*), assuming being risk set at time *t*. Marginal Cox models [3] can be used to estimate *k*-times event probability as the difference between the marginal probabilities of *k*^th^ and (*k* − 1)^th^ events.

During following up subjects, we often observe a terminal event, such as death, which preclude subsequent repeated events and induce informative censoring because of correlations between repeated events and a terminal event [4]. Thus, using joint frailty models [5–7], terminal events were regarded as informative censoring, and conditional distributions of repeated events on frailty could be obtained. This modeling of repeated events adjusted for the correlation between repeated events and a terminal event may be difficult to interpret clinically. Instead, a comprehensive approach should consider probabilities or hazards of both repeated and terminal events that are subject to estimation. In this context, numbers of repeated and terminal events are regarded as semi-competing risks [8, 9] that encompass an illness–death process [10]. Our proposed method is based on the illness–death process and dynamically predicts the probability of terminal events.

Researcher might include longitudinal data such as biomarkers, health status, and disease histories as time-dependent covariates in a prediction model. These clinical measures are usually internal covariates and required extra modeling to predict survival functions accurately [11, 12], and joint modeling and landmarking are major approaches and explored the predictive performance [13–15]. The former couples longitudinal trajectory and survival models [16–20], and for continuous variables observed at some interval; the model explicitly specifies its trajectory for accurate predictions. The latter landmarking approach is robust to model misspecification for longitudinal processes [21–24] and uses only longitudinal data *Z*(*s*) accumulated until a certain fixed (landmarking) time *s*. This procedure treats longitudinal data as fixed external covariates and leads to adequate modeling for time-dependent internal covariates. Recently parsimonious modeling approach in longitudinal data was proposed [25]. Landmarking models of residual lifetimes *t* – *s* have been developed [21, 26], and competing risks data have been considered in landmarking models after extension based on Fine-Gray [27] models [28] and on dynamic pseudo-observations (DPOs) [29].

Mauguen et al. [18] and Musoro et al. [24] examined dynamic prediction on repeated events data. The former was interested in dynamic prediction of death using cancer relapse, which is repeated events data. Because of the dependency of death and relapse, time to death and time to relapse were linked by joint frailty term. The latter was interested in the dynamic prediction of infection risk using longitudinal marker and history of infection. Because patients repeat infection events, Cox frailty model was employed and landmarking technique was used for the longitudinal marker. The above methods cannot be used as they are in situations where the risk of each number of repeated event and the risk of a terminal event are predicted in parallel. Therefore, we propose a prediction method using DPOs for repeated events data in the presence and absence of terminal events.

DPOs are proposed in the framework of pseudo-observations, which are each subject’s contribution to occurring the event replace each observed or censored event indicator at some time point. Although the realized value of event indicator is unknown on the censored subject, the contribution can be calculated by jackknife estimates. So covariate information on a censored subject can be incorporated into a generalized linear model directly. Unlike multiple imputations, it is unnecessary of pseudo-observations to repeat the creation of datasets nor combine results obtained from datasets. DPOs are extended such pseudo-observations in dynamic prediction for competing risks [29], and the idea of DPOs was applied to illness-death process [30]. So our proposal is regarded as an extension of DPOs for semi-competing risks settings, and that provides a dynamic prediction method in the presence of a terminal event. It is unnecessary to specify the correlation between repeated events and a terminal event, or model hazard function addressed by existing methods mainly. Although the asymptotic behavior of this approach has been demonstrated [29, 31–33], few studies have assessed the performance of pseudo-observations in finite samples. Thus, we conducted simulations to evaluate bias and efficiency of DPOs. Finally, we applied the proposed method to data of Japanese patients with colorectal liver metastases.

## Method

### Dynamic prediction and landmarking

For subject *i* (*i* = 1, …, *n*), let *T*_*ij*_, *T*_*i*_^D^, and *C*_*i*_ be times for *j*^th^ (*j* = 1, 2, …, *J*_*i*_) repeated events, a terminal event and censoring time, respectively, and let ***Z***_*i*_(*t*) = [*Z*_*i*1_(*t*), …,*Z*_*i*p_(*t*)]^T^ be the time-dependent covariate vector at time *t*. Therefore, potential data {{*T*_*ij*_}, *T*_*i*_^D^, *C*_*i*_, ***Z***_*i*_(*t*)} in subject *i* are assumed to be independent from {{*T*_*i’j*_}, *T*_*i’*_^D^, *C*_*i’*_, ***Z***_*i’*_(*t*)} in another subject (*i’*) (assumption 1). If a terminal event is not considered, *T*_*i*_^*D*^ is set to ∞. Because dynamic prediction estimates a conditional probability of an event based on at risk at a certain time point *s*, we refined notations of random variables of repeated events time as follows: Let ${T}_{mik}^{\ast }$ be *k*^th^ (*k* = 1, 2, …) repeated event time counted from *m*^th^ (*m* = 0,…, *M*) conditional(landmark) time *s*_*m*_. For example, if the subject *i* has experienced two events until *s*_*m*_, time ${T}_{mi1}^{\ast },{T}_{mi2}^{\ast },{T}_{mi3}^{\ast }$ is assigned to *T*_*i*3_, *T*_*i*4_, *T*_*i*5_, respectively. For parsimonious definition, the number of events occurred before *s*_*m*_ could be treated as covariates *Z*(*s*) or stratified factor to taking into consideration for dynamic prediction. We would illustrate the former approach with a real example in section 2.5. We abbreviate *s*_*m*_ and ${T}_{mik}^{\ast }$ to *s* and ${T}_k^{\ast }$, respectively.

Hence, the probability of just *k*-time events occurring within (*s*, *s* + *w*) and a terminal event occurring after *s* + *w* given the subject being at risk at time *s* is expressed as follows:

1 $$ {F}_k\left(s+w|s\right)=\Pr \left({T}_k^{\ast}\le s+w,{T}_{k+1}^{\ast }>s+w,{T}^D>s+w\left|{T}^D>s\right.\right),\kern0.36em \mathrm{for}\;k=0,1,\dots, $$

and the probability of a terminal event is expressed as follows:

2 $$ {F}^D\left(s+w|s\right)=\Pr \left({T}^D\le s+w|{T}^D>s\right), $$

where *w* (*w* > 0) is the prediction window. To make these probabilities {*F*^*D*^, *F*_0_, *F*_1_, …} mutually exclusive and equal to a total of 1, we define dynamic prediction to estimate conditional probabilities {*F*^*D*^, *F*_0_, *F*_1_, …} and ‘at risk’ as the status of subjects who have experienced neither censoring nor terminal events.

### Proposed DPOs

The predicted probabilities presented in eq.(1) and eq.(2) are regarded as expectations of event indicators. For example, $E\left\{I\left({T}_k^{\ast}\le s+w,{T}_{k+1}^{\ast }>s+w\left|{T}^D>s\right.\right)\right\}$ represents the probability of *k*-times repeated events. If there are no censored subjects, this expectation can be calculated as the average of indicators for each subject. Conversely, these indicators must be unknown for the censored subject. Andersen et al. proposed that indicators among all subjects could be replaced with pseudo-observations [34], and Nicolaie et al. extended them to dynamic predictions named dynamic pseudo-observations (DPOs) [29]. Pseudo-observations are calculated by the difference between the estimates of predicted probability multiplied by sample size *n* and the leave-one-out estimates multiplied by *n*-1, and looked upon as predicted indicators if censoring does not occur in any subjects. In addition to this, predicted probabilities can be calculated from the generalized linear model with regression pseudo-observations on some covariates, directly.

For subject *i* at risk at landmark time *s*, DPOs of *k-*times repeated events and the terminal event were defined as follows, respectively:

3 $$ {\widehat{\theta}}_{ik}(s)={n}_s\cdotp {\widehat{F}}_k\left(s+w|s\right)-\left({n}_s-1\right)\cdotp {\widehat{F}}_k^{\left(-i\right)}\left(s+w|s\right),\mathrm{for}\;k=0,1,\kern0.5em \dots, $$

4 $$ {\widehat{\theta}}_i^D(s)={n}_s\cdotp {\widehat{F}}^D\left(s+w|s\right)-\left({n}_s-1\right)\cdotp {\widehat{F}}^{D,\left(-i\right)}\left(s+w|s\right), $$

where *n*_*s*_ is the number of risk sets at the landmark time *s*, and ${\widehat{F}}_k$ and ${\widehat{F}}^D$ are consistency estimates of *F*_*k*_ and *F*^*D*^, respectively, as described in 2.2.1 and 2.2.2. The superscript (−*i*) represents corresponding leave-one-out estimates from datasets that are omitted only for subject *i*.

We have added the following assumptions that underlie the landmark dataset.

- Censoring time *C*_*i*_ is independent on potential event times {T^*^_ij_}, *T*_*i*_^D,^ and covariates ***Z***_*i*_(*s*); noninformative censoring (assumption 2)
- *G*(·) denotes the survival function of censoring. Hence, for any τ; τ > *s* + *w*, *G*(τ) > 0. (assumption 3)

From previous researches [29, 31], assumptions 1–3 were required for the DPOs listed in sections DPOs in the absence of a terminal event and DPOs in the presence of a terminal event to maintain consistency of estimated regression coefficients and corresponding variances, as presented in section Construction of the dynamic prediction regression model.

#### DPOs in the absence of a terminal event

**Fig. 1:**
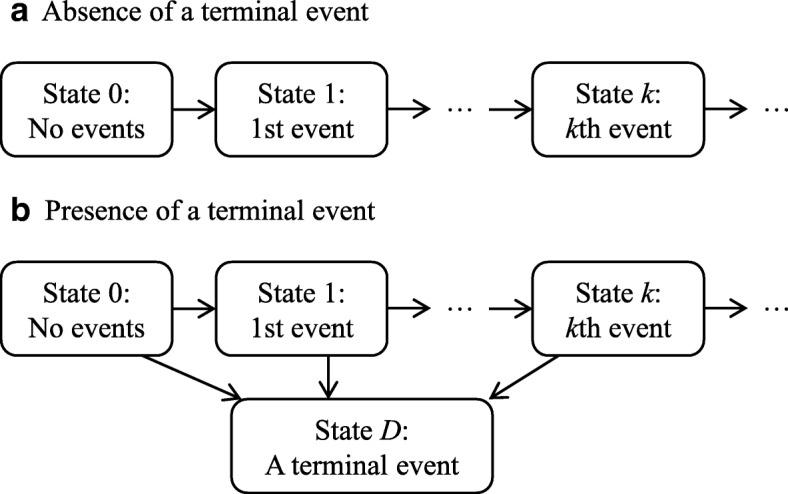
Assumed multi-state model for repeated events processes; (**a**) when no terminal event was assumed, (**b**) when a terminal event was assumed

In the presence of repeated events only, a terminal event may be treated as usual censoring, and we target the probability in eq.(1). Assuming the multi-state model in repeated events process (Fig. 1a), prediction probabilities can be obtained using the product–integral as the state occupation probability (detailed in Additional file 1: Appendix). Under Markov processes between states, this transition probability matrix is termed Aalen–Johansen (AJ) estimator [35] and has consistency in *O*(*n*^1/2^) with assumptions 1,2 and some regulatory conditions [36]. Furthermore, Datta and Satten [37] showed that empirical state occupation probabilities for the whole dataset could be consistently estimated using the product integral, in the same manner as that using the AJ estimator. Hence, the estimated transition matrix using product integral is referred to as the AJ estimator regardless of Markovian principles.

Because all subjects belong to state 0 (initial state) at the landmark point in the multi-state model for repeated events (Fig. 1a), the probability of the *k*^th^ repeated event is denoted by *P*_0*k*_(*t*), which is the transition probability from state 0 to state *k* pictured in Fig. 1a. Accordingly, DPOs for the *k-*time event in eq.(3) are calculated as follows:

5 $$ {\widehat{\theta}}_{ik}(s)={n}_s\cdotp {\widehat{P}}_{0k}\left(s,s+w\right)-\left({n}_s-1\right)\cdotp {\widehat{P}}_{0k}^{\left(-i\right)}\left(s,s+w\right) $$

This method is referred to as the DPOs-based on the AJ estimator.

The method described in eq.(5) requires difficult matrix computation because of the product integral. Thus, to improve convenience, we propose another DPO using the sequence of repeated events and rewrite the *k*-times event probability function *F*_*k*_(*s* + *w*|*s*) as follows:

$$ {\displaystyle \begin{array}{c}{F}_k\left(s+w\left|s\right.\right)=E\left\{I\left({T}_k^{\ast}\le s+w,{T}_{k+1}^{\ast }>s+w\right)\right\}\\ {}=E\left[\left\{1-I\left({T}_k^{\ast }>s+w\right)\right\}\cdotp I\left({T}_{k+1}^{\ast }>s+w\right)\right]\\ {}=E\left\{I\left({T}_{k+1}^{\ast }>s+w\right)-I\left({T}_k^{\ast }>s+w\right)\right\}\\ {}=E\left\{I\left({T}_{k+1}^{\ast }>s+w\right)\right\}-E\left\{I\left({T}_k^{\ast }>s+w\right)\right\}\\ {}={S}_{k+1}^{\ast}\left(s+w\right)-{S}_k^{\ast}\left(s+w\right)\end{array}} $$

where ${S}_k^{\ast}\left(\cdotp \right)$ is survival function for ${T}_k^{\ast }$. The probability of the *k-*times event is expressed as the difference between the survival probability of the (*k* + 1)^th^ event and the *k*^th^ event. Subsequently, ${S}_k^{\ast}\left(\cdotp \right)$ can be directly and consistently estimated using Kaplan–Meier (KM) estimators [38] or using the exponential transformed Nelson–Aalen estimator in *O*(*n*^1/2^). However, we only consider using the KM estimator to estimate ${S}_k^{\ast}\left(\cdotp \right)$, and DPOs based on KM estimator was calculated as follows:

6 $$ {\widehat{\theta}}_{ik}(s)={n}_s\cdotp \left\{{\widehat{S}}_{k+1}^{\ast}\left(s+w\right)-{\widehat{S}}_k^{\ast}\left(s+w\right)\right\}-\left({n}_s-1\right)\cdotp \left\{{\widehat{S}}_{k+1}^{\ast, \left(-i\right)}\left(s+w\right)-{\widehat{S}}_k^{\ast, \left(-i\right)}\left(s+w\right)\right\} $$

#### DPOs in the presence of a terminal event

The illness-death model is applicable to situations with only one terminal event and one non-terminal event. As an extension of the illness-death model, the multi-state model shown in Fig. 1(b) expresses repeated event processes in the presence of a terminal event. Accordingly, DPOs for repeated events are as in eq.(5), and DPOs for a terminal event in eq.(4) are described as follows:

7 $$ {\widehat{\theta}}_i^D(s)={n}_s\cdotp {\widehat{P}}_{0D}\left(s,s+w\right)-\left({n}_s-1\right)\cdotp {\widehat{P}}_{0D}^{\left(-i\right)}\left(s,s+w\right). $$

### Construction of the dynamic prediction regression model

To demonstrate dynamic prediction at one fixed landmark time *s*, the relationships between event probabilities and landmarking covariates **Z**_*i*_(*s*) are constructed in the generalized linear model framework. The fixed landmark regression model is described as follows:

$$ g\left\{{\boldsymbol{\uptheta}}_i(s)\right\}=\boldsymbol{\upbeta} {(s)}^{\top }{\mathbf{Z}}_i^{\ast }(s) $$

where *g* denotes a link function. Accordingly, we regard ${\boldsymbol{\uptheta}}_i(s)={\left[{\theta}_{i1}(s),{\theta}_{i2}(s),\cdots, {\theta}_{iJ_s}(s)\right]}^{\top }$ as multinomial probability and exclusion of *θ*_*i*0_(*s*) from **θ**_*i*_(*s*) evaded parameter redundancy. Note that ${\mathbf{Z}}_i^{\ast }(s)$ is among baseline and longitudinal covariates up to the landmark time *s* and the intercept. For example, when we assume a multinomial model [39, 40] with a generalized logit link to DPOs, the link function is logit and covariates ${\mathbf{Z}}_i^{\ast }(s)$ are formed as follows:

$$ {\mathbf{Z}}_i^{\ast }(s)=\left(\begin{array}{cccc}1\kern0.5em {\mathbf{Z}}_i{(s)}^{\top }& & & 0\\ {}& 1\kern0.5em {\mathbf{Z}}_i{(s)}^{\top }& & \\ {}& & \ddots & \\ {}0& & & \begin{array}{cc}1& {\mathbf{Z}}_i{(s)}^{\top}\end{array}\end{array}\right). $$

The regression coefficient vector **β** is calculated by solving the following estimating equation:

$$ \mathbf{U}\left\{\boldsymbol{\upbeta} (s)\right\}=\sum \limits_i{\mathbf{D}}_i{(s)}^{\top }{\mathbf{V}}_i^{-1}(s)\left\{{\widehat{\boldsymbol{\uptheta}}}_i(s)-{\boldsymbol{\uptheta}}_i(s)\right\}=0,\kern1em {\mathbf{D}}_i(s)=\frac{\partial {\boldsymbol{\uptheta}}_i(s)}{\partial \boldsymbol{\upbeta} (s)}, $$

where **V**_*i*_(*s*) follows a multinomial distribution. Accordingly, asymptotic variance of **β**(*s*) is calculated from model variance as $\mathrm{Var}\left\{\widehat{\boldsymbol{\upbeta}}(s)\right\}={\left\{\sum \limits_i{\widehat{\mathbf{D}}}_i{(s)}^{\top }{\widehat{\mathbf{V}}}_i^{-1}(s){\widehat{\mathbf{D}}}_i(s)\right\}}^{-1}$. Estimation in event probability is transformed from $\widehat{\boldsymbol{\upbeta}}(s)$ using the inverse link function, and its variance is calculated from $\mathrm{Var}\left\{\widehat{\boldsymbol{\upbeta}}(s)\right\}$ using the delta method.

Because predicted probabilities at each time point were estimated from different fixed landmark regression models, predicted probabilities tend to vary with proceeding landmark times. Especially when the number at risk is small, predicted probabilities take a large value change. Hence, to enhance an interpretation, the smoothers *f*(*s*) against landmark times are included in regression coefficients to continuously predict event probabilities. In addition, the precision of predicted probabilities is likely improved because the information from several landmark times can be used. This *supermodel* is described that regression coefficients are divided into polynomial smoother parts and invariant parts and are multiplied by **β**(*s*) = *f*(*s*) · **β**. In practice, the following covariates are stacked for several landmark times, and their interactions are described in the following polynomial function *f*_0_(*s*), *f*_1_(*s*), ⋯, *f*_*h*_(*s*),

$$ {\tilde{\mathbf{Z}}}_i^{\ast }=\left(\begin{array}{ccc}{f}_0\left({s}_0\right){\mathbf{Z}}_i^{\ast}\left({s}_0\right)& \cdots & {f}_h\left({s}_0\right){\mathbf{Z}}_i^{\ast}\left({s}_0\right)\\ {}& \vdots & \\ {}{f}_0\left({s}_M\right){\mathbf{Z}}_i^{\ast}\left({s}_M\right)& \cdots & {f}_h\left({s}_M\right){\mathbf{Z}}_i^{\ast}\left({s}_M\right)\end{array}\right),{f}_0\left(\cdotp \right)=1. $$

Similarly, DPOs are stacked as ${\widehat{\boldsymbol{\uptheta}}}_i={\left[{\widehat{\boldsymbol{\uptheta}}}_i{\left({s}_0\right)}^{\top },\cdots, {\widehat{\boldsymbol{\uptheta}}}_i{\left({s}_0\right)}^{\top}\right]}^{\top }$. However, because multiple DPOs from the same subject are present, a generalized estimating equations approach [41] was adapted and solved as follows:

$$ \mathbf{U}\left(\tilde{\boldsymbol{\upbeta}}\right)=\sum \limits_i{{\tilde{\mathbf{D}}}_i}^{\top }{\tilde{\mathbf{V}}}_i^{-1}\left\{{\widehat{\boldsymbol{\uptheta}}}_i-{\boldsymbol{\uptheta}}_i\right\}=0,\kern1em {\tilde{\mathbf{D}}}_i=\frac{\partial {\boldsymbol{\uptheta}}_i}{\partial \tilde{\boldsymbol{\upbeta}}}. $$

The robust (sandwich) estimator is used for the covariance matrix of $\tilde{\boldsymbol{\upbeta}}$ and only specified as the independent type because of consistencies of estimating equations [42].

### Simulation studies

Because the above asymptotic properties of DPOs were already shown, to assess the performance of proposed DPOs in a finite and realistic sample (*n* = 100), we conducted Monte Carlo simulation studies. Conditions WITHOUT/WITH terminal events are described in Predictions of repeated events without a terminal event and Prediction of repeated events WITH a terminal event, respectively.

#### Predictions of repeated events without a terminal event

Repeated events for subject *i* were limited to two, and corresponding times were represented as *t*_*i*1_ and *t*_*i*2_. Subsequent exponential distribution with hazards *λ*_*i*1_ and *λ*_*i*2_ resulted in the first event time *t*_*i*1_ and the lag time between the first and second time *t*_*i*2_ − *t*_*i*1_ as follows:

$$ {\lambda}_{i1}={u}_i{\lambda}_{01},\kern1em {\lambda}_{i2}={u}_i{\lambda}_{02}, $$

where *u*_*i*_ denotes the frailty parameter, and *λ*_01_ and *λ*_02_ are baseline hazards. To assume heterogeneity of event occurrence between subjects, *u*_*i*_ must follow a gamma distribution with shape and rate parameters of 0.5. Moreover, the correlation between *t*_*i*1_ and *t*_*i*2_ − *t*_*i*1_ is theoretically 0.5 according to Kendall’s tau, and when *u*_*i*_ is constant at 1, no heterogeneity is present. Baseline hazards *λ*_01_ and *λ*_02_ are set to reflect Markov or semi-Markov process. In particular, *λ*_01_ = *λ*_02_ = 1 under the assumption of Markovian event processes. When *λ*_01_ = 1 and *λ*_02_ = 2, a semi-Markov process is assumed, such that the first event accelerates the hazard of the second event. Finally, probabilities for each number of events at time 1 were calculated from generated data (*n* = 100), and empirical probabilities were considered to be *true values* for each replication.

Right censoring was generated using an exponential distribution that is independent of repeated event processes, with hazards *λ*_*c*_ = 0.5 or *λ*_*c*_ = 2. Proposed DPOs based on the AJ (eq.(5)) and KM (eq.(6)) estimators were applied to these right-censored data, and expectations of these DPOs were calculated as *dynamic predicted values*. In all scenarios, bias, and efficiency of prediction at *t* = 0 with window *w* = 1 were evaluated from 1000 repetitions of true and dynamic predicted values using the absolute bias, and the root mean squared error (RMSE), respectively.

#### Prediction of repeated events WITH a terminal event

To examine the performance of DPOs in eq.(5) and eq.(7) in the presence of terminal events, we performed simulation studies as described in section Predictions of repeated events without a terminal event. In addition to the two repeated event times, the potential time for the occurrence of a terminal event *t*_*iD*_ was generated using the following exponential distribution with hazard *λ*_*iD*_:

$$ {\lambda}_{i1}={u}_i{\lambda}_{01},\kern1em {\lambda}_{i2}={u}_i{\lambda}_{02},\kern1em {\lambda}_{iD}={u}_i{\lambda}_{0D}, $$

where *λ*_0*D*_ is a baseline hazard and is fixed at 0.3. The frailty parameter *u*_*i*_ was subject to a gamma distribution; therefore, correlations of any pair among *t*_*i*1_, *t*_*i*2_ − *t*_*i*1_ and *t*_*D*_ were of the same strength. Other procedures were similar to those described in section Predictions of repeated events without a terminal event.

### Application of the DPOs to colorectal liver metastases data

We applied the proposed DPOs method to colorectal liver metastases data of 263 patients from Japan [1]. The database had been prospectively collected from 263 patients who underwent upfront hepatic resections from January 1996 to December 2010 at the Hepato-Biliary-Pancreatic Surgery Division, Department of Surgery, Graduate School of Medicine, The University of Tokyo. No included patient had received postoperative adjuvant chemotherapy or was enrolled in clinical trials. A total of 202 patients (76.8%) suffered first recurrences and 108 (53.3%) of these were re-resected. Patients had up to four recurrences, and dynamic predictions of 3-year event risks were calculated using information from the most recent tumor to identify patients at high risk of recurrence.

In these analyses, 3-year risks of recurrence were classified as no recurrence, single recurrence or multiple recurrences, and were dynamically predicted according to numbers of recurrences (≥1 vs. 0), numbers of tumors (single or multiple) and tumor lengths (> 2 cm or ≤ 2 cm) before landmark time. Initially, DPOs from eq.(5) and eq.(6) were applied, and death was thought as censoring. In addition to the fixed landmark regression model, a supermodel was constructed using cubic smoothers against landmark time as follows: **f**(*s*) = [*f*_0_(*s*),  … , *f*_3_(*s*)] = [1, *s*, *s*^2^, *s*^3^].

To take a terminal event into consideration, 3-year risks of recurrence were classified as no recurrence, single recurrence, multiple recurrence or death. DPOs in eq.(5) and eq.(7) were applied and a fixed landmark model and a supermodel with cubic smoothers against landmark time were constructed.

## Results

### Simulation

#### Performance of DPOs in the absence of a terminal event

To examine scenario characteristics, the mean *true* probability of event numbers, censored proportions and Kendall’s tau between *t*_*i*1_ and *t*_*i*2_ − *t*_*i*1_ were calculated (Table 1). In all scenarios, true probabilities of event numbers were > 10%, and event times were censored heavily at *λ*_*c*_ = 2 and could be observed the event time at < 25% subject.

**Table 1:** Summary of data generated in the absence of a terminal event

Simulation parameters				True proportion of event numbers			Censored proportion	Kendall’s tau between *t*_*i*1_ and *t*_*i*2_ − *t*_*i*1_
*u*_*i*_	*λ*_01_	*λ*_02_	*λ*_*c*_	No events	1	2		
1	1	1	0.5	36.7	36.8	26.5	35.7	−0.001
1	1	2	0.5	36.7	23.3	40.0	33.4	−0.001
Γ(0.5,0.5)	1	1	0.5	57.5	19.4	23.1	35.2	0.497
Γ(0.5,0.5)	1	2	0.5	57.5	13.1	29.4	33.7	0.497
1	1	1	2	36.7	36.8	26.5	81.2	−0.001
1	1	2	2	36.7	23.4	39.9	77.5	−0.001
Γ(0.5,0.5)	1	1	2	57.4	19.6	26.0	79.8	0.496
Γ(0.5,0.5)	1	2	2	57.4	13.4	29.2	77.0	0.496

Absolute bias and RMSE values are presented in Table 2. In all scenarios, both methods resulted in approximately zero bias indicating accurate estimates of event processes based on the use of Markovian or semi-Markovian principles and heterogeneity of event occurrences between subjects. RMSE in the method based on the AJ estimator was less than or equally efficacious compared with the method based on the KM estimator, and RMSE ratios were 0.9–1.0.

**Table 2:** Simulation results in the absence of a terminal event

Scenario				DPOs based on AJ estimator^a^			DPOs based on KM estimator^b^		
*u*_*i*_	*λ*_01_	*λ*_02_	*λ*_*c*_	no events	1 event	2 events	No events	1 event	2 events
Absolute bias^c^									
1	1	1	0.5	−0.0005	0.0004	0.0001	−0.0005	0.0008	−0.0004
1	1	2	0.5	−0.0005	0.0005	−0.0001	−0.0005	0.0011	−0.0006
Γ(0.5, 0.5)	1	1	0.5	−0.0006	0.0004	0.0002	−0.0006	0.0003	0.0003
Γ(0.5, 0.5)	1	2	0.5	−0.0006	0.0006	−0.00004	−0.0006	0.0007	−0.0001
1	1	1	2	−0.0034	0.0086	−0.0052	−0.0044	0.0102	−0.0063
1	1	2	2	−0.0057	0.0105	−0.0048	−0.0067	0.0127	−0.0066
Γ(0.5, 0.5)	1	1	2	−0.0066	0.0112	−0.0047	−0.0066	0.0088	−0.0022
Γ(0.5, 0.5)	1	2	2	−0.0113	0.0174	−0.0061	−0.0113	0.0129	−0.0016
Root Mean Squared Error (RMSE)									
1	1	1	0.5	0.0282	0.0340	0.0270	0.0282	0.0347	0.0280
1	1	2	0.5	0.0282	0.0304	0.0281	0.0282	0.0328	0.0310
Γ (0.5, 0.5)	1	1	0.5	0.0260	0.0264	0.0220	0.0260	0.0284	0.0243
Γ (0.5, 0.5)	1	2	0.5	0.0260	0.0241	0.0245	0.0260	0.0268	0.0270
1	1	1	2	0.0837	0.0969	0.0733	0.0851	0.1004	0.0758
1	1	2	2	0.0840	0.0935	0.0813	0.0855	0.0997	0.0852
Γ (0.5, 0.5)	1	1	2	0.0702	0.0762	0.0606	0.0703	0.0797	0.0641
Γ (0.5, 0.5)	1	2	2	0.0695	0.0693	0.0628	0.0696	0.0753	0.0682

^a^ Proposed in eq.(5)

^b^ Proposed in eq.(6)

^c^ Mean differences between *true values* and *dynamic predicted values*; *True values* are empirical probabilities of event numbers calculated from potential event times. *Dynamic predicted values* are the expectations of proposed DPOs

#### Performance of DPOs in the presence of a terminal event

Scenario characteristics are shown in Table 3. Results of absolute bias and RMSE analyses are shown in Table 4. Little bias was observed, and RMSE values were consistent with those described in section 3.1.1.

**Table 3:** Summary of data generated in the presence of a terminal event

Simulation parameters					True proportion of event numbers				Censored proportion	Kendall’s tau		
*u*_*i*_	*λ*_01_	*λ*_02_	*λ*_0 *D*_	*λ*_*c*_	No events	1	2	terminal		(*t*_*i*1_, *t*_*i*2_ − *t*_*i*1_)	(*t*_*i*1_, *t*_*i*D_)	(*t*_*i*2_ − *t*_*i*1_, *t*_*D*_)
1	1	1	0.3	0.5	27.3	27.2	19.5	26.0	34.1	−0.0003	0.002	0.001
1	1	2	0.3	0.5	27.3	17.1	29.5	26.0	34.1	−0.0003	0.002	0.001
Γ(0.5,0.5)	1	1	0.3	0.5	52.7	14.4	11.8	21.1	35.2	0.501	0.500	0.499
Γ(0.5,0.5)	1	2	0.3	0.5	52.7	10.3	16.0	21.1	35.2	0.501	0.500	0.499
1	1	1	0.3	2	27.2	27.2	19.6	26.9	77.9	−0.001	0.0003	0.002
1	1	2	0.3	2	27.2	17.4	29.5	26.9	77.7	−0.001	0.001	0.004
Γ(0.5,0.5)	1	1	0.3	2	52.3	14.7	12.2	20.9	78.6	0.499	0.498	0.497
Γ(0.5,0.5)	1	2	0.3	2	52.2	10.8	16.2	20.9	78.5	0.497	0.495	0.495

**Table 4:** Simulation results in the presence of a terminal event

Scenario					DPOs based on AJ estimator^a^			
*u*_*i*_	*λ*_01_	*λ*_02_	*λ*_0 *D*_	*λ*_*c*_	no events	1 event	2 events	a terminal event
Absolute bias^b^								
1	1	1	0.3	0.5	0.0003	− 0.00002	0.0006	−0.0008
1	1	2	0.3	0.5	0.0003	−0.0004	0.0010	−0.0008
Γ (0.5, 0.5)	1	1	0.3	0.5	0.0011	−0.0002	− 0.0011	0.0002
Γ (0.5, 0.5)	1	2	0.3	0.5	0.0011	−0.0002	− 0.0010	0.0002
1	1	1	0.3	2	−0.0025	−0.0017	0.0040	0.0002
1	1	2	0.3	2	−0.0057	0.0088	−0.0032	0.0001
Γ (0.5, 0.5)	1	1	0.3	2	−0.0081	0.0086	0.0055	−0.0060
Γ (0.5, 0.5)	1	2	0.3	2	−0.0094	0.0137	0.0010	−0.0053
Root Mean Squared Error (RMSE)								
1	1	1	0.3	0.5	0.0264	0.0316	0.0241	0.0245
1	1	2	0.3	0.5	0.0264	0.0270	0.0281	0.0245
Γ (0.5, 0.5)	1	1	0.3	0.5	0.0261	0.0238	0.0207	0.0211
Γ (0.5, 0.5)	1	2	0.3	0.5	0.0261	0.0214	0.0225	0.0209
1	1	1	0.3	2	0.0724	0.0836	0.0697	0.0681
1	1	2	0.3	2	0.0704	0.0749	0.0783	0.0687
Γ (0.5, 0.5)	1	1	0.3	2	0.0703	0.0644	0.0526	0.0562
Γ (0.5, 0.5)	1	2	0.3	2	0.0701	0.0609	0.0620	0.0575

^a^ Proposed in eq.(5) and eq.(7)

^b^ Mean differences between *true values* and *dynamic predicted values*; *True values* are empirical probabilities of event numbers calculated using potential event times. *Dynamic predicted values* are expectations of proposed DPOs

### Application of the DPOs to a real example

First, we applied two-types DPOs described in eq.(5) and eq.(6). Parameter estimates from the supermodel are shown in Table 5, and few differences in parameter estimates from the two types of DPOs were observed. Predicted probabilities are shown in Additional file 2.

**Table 5:** Parameter estimates in landmarking supermodel using two-types of dynamic pseudo observations (DPOs)

	DPOs based on AJ estimator (eq.(5))				DPOs based on KM estimator (eq.(6))			
	1 recurrence		2 or more recurrences		1 recurrence		2 or more recurrences	
	estimate	robust s.e.	estimate	robust s.e.	estimate	robust s.e.	estimate	robust s.e.
Intercept	−1.22	1.99	1.30	4.27	−1.06	1.97	−0.13	4.23
Time (year)								
*s*	2.19	3.28	−6.24	6.28	1.92	3.24	−3.98	6.15
*s*^2^	−1.53	1.64	2.42	2.69	−1.41	1.62	1.38	2.61
*s*^3^	0.22	0.24	−0.32	0.34	0.21	0.24	−0.19	0.33
The number of recurrences (1 or more / 0)								
Intercept	1.92	2.12	0.49	4.72	1.84	2.09	1.51	4.54
*s*	−2.87	3.26	3.36	7.01	−2.70	3.21	1.78	6.73
*s*^2^	1.65	1.55	−2.26	2.99	1.58	1.53	−1.55	2.87
*s*^3^	−0.24	0.22	0.38	0.38	−0.23	0.22	0.29	0.37
Multiple tumors / single tumor								
Intercept	4.46	2.09	−2.27	4.32	4.31	2.05	−1.30	4.44
*s*	−7.87	3.21	3.16	6.64	−7.57	3.15	1.38	6.77
*s*^2^	3.86	1.50	−0.91	2.89	3.71	1.48	0.01	2.91
*s*^3^	−0.53	0.21	0.09	0.37	−0.51	0.21	−0.04	0.36
The length of tumor (> 2 cm / ≤2 cm)								
Intercept	−0.10	1.87	−1.19	4.07	−0.09	1.89	−0.66	4.00
*s*	0.31	2.88	4.08	6.21	0.30	2.91	3.29	6.04
*s*^2^	−0.21	1.34	−2.05	2.70	−0.19	1.37	−1.74	2.58
*s*^3^	0.03	0.19	0.29	0.35	0.02	0.20	0.26	0.33

**Fig. 2:**
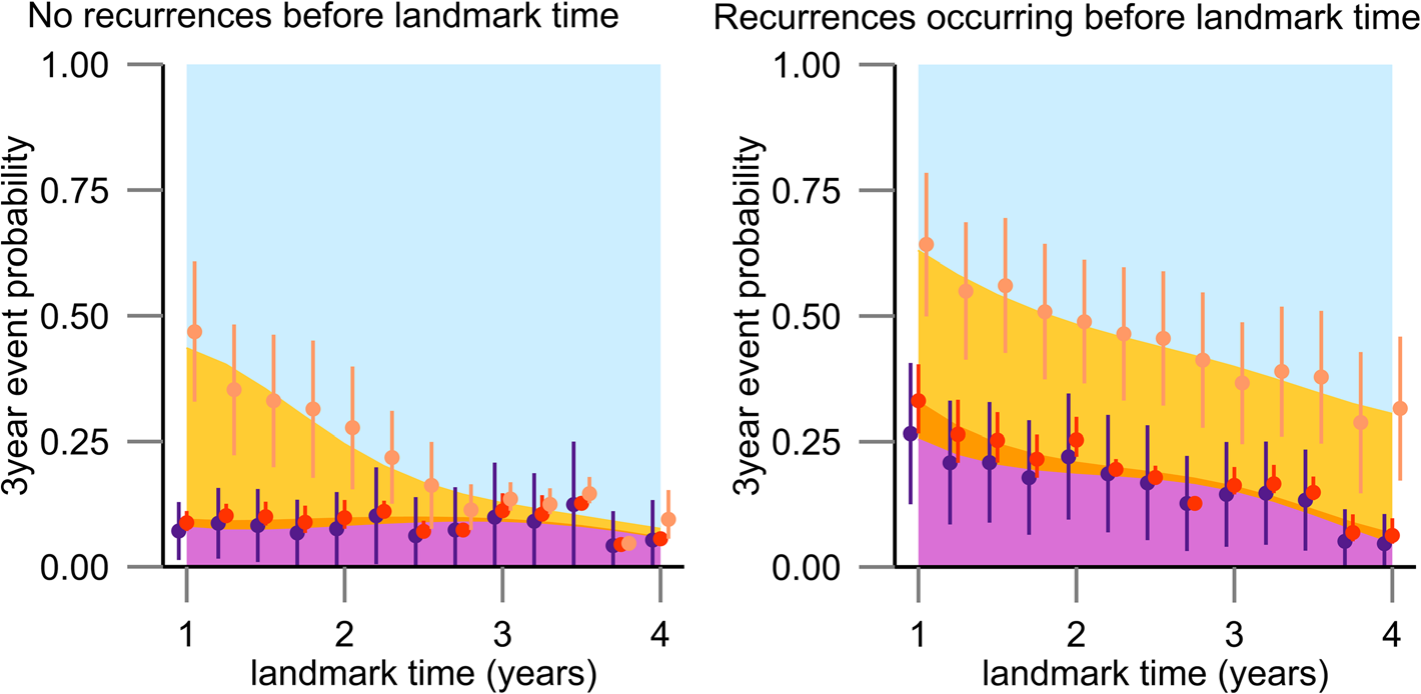
Stacked 3-year event probability in subjects with single tumors of ≤ 2 cm. Stacked graphs show predicted risks of no recurrence for 3 years after landmark time (blue), 1 time recurrence (yellow), ≥2 recurrence (orange) and death (purple). Circles and error bars show point estimates and 95% CI, respectively, from the fixed landmark regression model. Filled areas show point estimates from the supermodel

Further, we applied DPOs that included terminal events. Predicted probabilities among patients with data for single and small (≤2 cm) tumors are shown in Fig. 2, and other predicted probabilities and parameter estimates of the supermodel are shown in Additional file 2. Patients who had experienced recurrences before landmark time had higher risks of recurrence and death than those who have not experienced recurrences, whereas among patients with single and small tumors at the first hepatic resection and no recurrence for approximately 3 years, subsequent recurrences were very rare. In contrast, patients with recurrences before landmark time had a moderate risk of re-recurrence. Also, multiple tumors resulted in worse prognoses compared with single tumors.

## Discussion

Here we applied dynamic prediction methods for repeated events using pseudo-observations and examined that performance using simulation studies. Prediction biases of the ensuing DPOs were calculated in a finite sample and indicated good performance regardless of processes for repeated events, which were assumed to be Markovian or semi-Markovian in the presence or absence of frailty. These assumptions were not testable using the observed data; therefore, independence of the present DPOs enables application of dynamic prediction based on landmarking to most types of time-to-event data observed in medical studies. Through real example, we drew the predicted probabilities of various type of events, such as repeated and terminal events. These comprehensive graphs must improve a subjective understanding of the disease.

In practice, it is easier to calculate proposed DPOs based on the KM estimator than on the AJ estimator using standard analysis packages because the AJ estimator requires matrix multiplication using proc. IML in SAS. A structure of landmark dataset and program codes of AJ estimator are shown in our website(https://github.com/yokotai/ and http://yokotai.wordpress.com/). Although repeated events can be modeled using marginal Cox model, DPOs are preferable to dynamic predictions for the following reasons: First, DPOs are free of the proportional hazards assumptions that are imposed on marginal Cox models. Second, generalized linear models with the supermodel can be used to smoothly predict probabilities against landmark time, whereas the supermodel of the marginal Cox modeling approach returns wiggly function of predicted probabilities against landmark time because of the step functions for estimates of baseline hazards. Since it is hard to think that the predicted probabilities repeat increasing and decreasing over time within a short time-span, it would be better to get the smooth curves for interpretation. Finally, the present DPOs have sufficient flexibility to accommodate the use of several link functions. Although a prediction model based hazard function is an analog of generalized linear model linked with complementary log-log function, our methods do not restrict any link function such as log, logit or probit.

Fitting DPOs to generalized linear models as multinomial outcomes may present practical issues because of the absence of standard analytic tools. Therefore, we recommend multivariate binary models after transformation using multinomial models to provide correct point estimates [39, 40]. Although variance estimates based on multivariate binary models have suspicious zero-covariance estimates between multinomial outcomes, these estimates can be used in practice because standard errors from binary models may slightly differ from those from multinomial models. In addition, because other covariates, model forms and selections of link function affect the lengths of confidence intervals, precision may be of less importance than accuracy in the prediction context.

There were two reasons why the simulation did not deal with the evaluation of predictive performance if we use longitudinal covariates available. First, model performance depends on specification of model form. On landmark supermodel, predicted probabilities at a certain time point was affected from another time point through smoothers *f*(*s*). This fact may cause more efficiency and more bias. The number of landmarking time and the interval of landmarking on a supermodel are worth investigating, but we have to make sure in a broad situation, and we would like to make it a future work. Second, there are few methods of predictive performance which can use for repeated events with terminal events. Model selection and model validation require the performance measures. We believe that loss function approaches with squared error, such as Brier scores in survival analyses [43, 44], should be applicable.

## Conclusion

In this article, we proposed a dynamic prediction method for repeated events data regardless of whether or not to consider a termination event. The method can predict the event probabilities consistently regardless of processes for repeated events, which were assumed to be Markovian or semi-Markovian in the presence or absence of frailty. Through a simulation study, the method works well in a relatively small finite sample. We contributed a new modeling method of repeated events data with a terminal event which provided predicted probabilities of his/her prognosis and had an intuitive interpretation.

## Additional files

Additional file 1: Appendix A short explanation of product-integral. (PDF 56 kb)

Additional file 2: Supplemental tables and figures of proposed DPOs to a real example. (PDF 885 kb)

## Abbreviations

AJ: Aalen-Johansen
DPO: Dynamic pseudo-observation
KM: Kaplan-Meier
RMSE: The root mean squared error
