# Supplementary material for: Dynamic prediction of repeated events data based on landmarking model: application to colorectal liver metastases data
Source: BMC Med Res Methodol. 2019 Feb 14;19:31. doi: 10.1186/s12874-019-0677-0 (PMC6376774; doi:10.1186/s12874-019-0677-0)
Supplement: Supplementary file 1 — Appendix A short explanation of product-integral. (PDF 56 kb) [file 12874_2019_677_MOESM1_ESM.pdf]

Appendix: Product-integral

Let $\lambda_{qr}(t)$ be the discrete hazard (intensity) function from state q to state r at time t, then, a nonparametric maximum likelihood estimator of $\lambda_{qr}(t)$ is calculated as $N_{qr}(t)/Y_q(t)$, where $N_{qr}(t)$ is the transition number from state q to state r and $Y_q(t)$ is the number of at risks in state q. The transition probability matrix $\mathbf{P}(t_0,t)=\left[P_{qr}(t_0,t)\right]$ can be estimated using a transition hazard matrix $\hat{\boldsymbol{\lambda}}(t)=\left[\hat{\lambda}_{qr}(t)\right]$ with $\hat{\lambda}_{qq}(t)=-\sum_{r:r\neq q}\hat{\lambda}_{qr}(t)$ as follows:

$$\hat{\mathbf{P}}(t_0,t)=\prod_{u:u\in(t_0,t]}\left\{\mathbf{1}+\hat{\boldsymbol{\lambda}}(u)\right\}$$

where $\prod$ is product integral and $\mathbf{1}$ is the identity matrix.
