# Supplementary material for: Dynamic prediction of repeated events data based on landmarking model: application to colorectal liver metastases data
Source: BMC Med Res Methodol. 2019 Feb 14;19:31. doi: 10.1186/s12874-019-0677-0 (PMC6376774; doi:10.1186/s12874-019-0677-0)

Table S1. Predict 3-year risks of recurrence regarding death as censoring.
(a) Parameter estimates in landmarking supermodel using dynamic pseudo observations (DPOs)

	DPOs based on AJ estimator (eq(6))				DPOs based on KM estimator (eq(7))			
	1 time		2 or more times		1 time		2 or more times	
	β	se.	β	se.	β	se.	β	se.
intercept	-1.22	1.99	1.30	4.27	-1.06	1.97	-0.13	4.23
time (year)								
s	2.19	3.28	-6.24	6.28	1.92	3.24	-3.98	6.15
s^2	-1.53	1.64	2.42	2.69	-1.41	1.62	1.38	2.61
s^3	0.22	0.24	-0.32	0.34	0.21	0.24	-0.19	0.33
the number of recurrences (1 or more / 0)								
intercept	1.92	2.12	0.49	4.72	1.84	2.09	1.51	4.54
s	-2.87	3.26	3.36	7.01	-2.70	3.21	1.78	6.73
s^2	1.65	1.55	-2.26	2.99	1.58	1.53	-1.55	2.87
s^3	-0.24	0.22	0.38	0.38	-0.23	0.22	0.29	0.37
multiple tumors / single tumor								
intercept	4.46	2.09	-2.27	4.32	4.31	2.05	-1.30	4.44
s	-7.87	3.21	3.16	6.64	-7.57	3.15	1.38	6.77
s^2	3.86	1.50	-0.91	2.89	3.71	1.48	0.01	2.91
s^3	-0.53	0.21	0.09	0.37	-0.51	0.21	-0.04	0.36
the length of tumor (>2cm / ≤2cm)								
intercept	-0.10	1.87	-1.19	4.07	-0.09	1.89	-0.66	4.00
s	0.31	2.88	4.08	6.21	0.30	2.91	3.29	6.04
s^2	-0.21	1.34	-2.05	2.70	-0.19	1.37	-1.74	2.58
s^3	0.03	0.19	0.29	0.35	0.02	0.20	0.26	0.33

β: parameter estimates; se.: robust standard error; s:smoothers in the super model.

(b) Predicted 3-year risks of recurrence

Stacked graphs are drawn the predicted risks of no recurrence for 3years after landmark time (blue), 1 time recurrence (yellow) and 2 or more times recurrence (orange). Circle and error bars show point estimates and 95% CI, respectively, by fixed landmark regression model. Filled areas show point estimates by supermodel. The left side figures show the results from DPOs based on AJ estimator and the right side figures show the results from DPOs based on KM estimator.

No recurrence before landmark time, single tumor not greater than 2cm

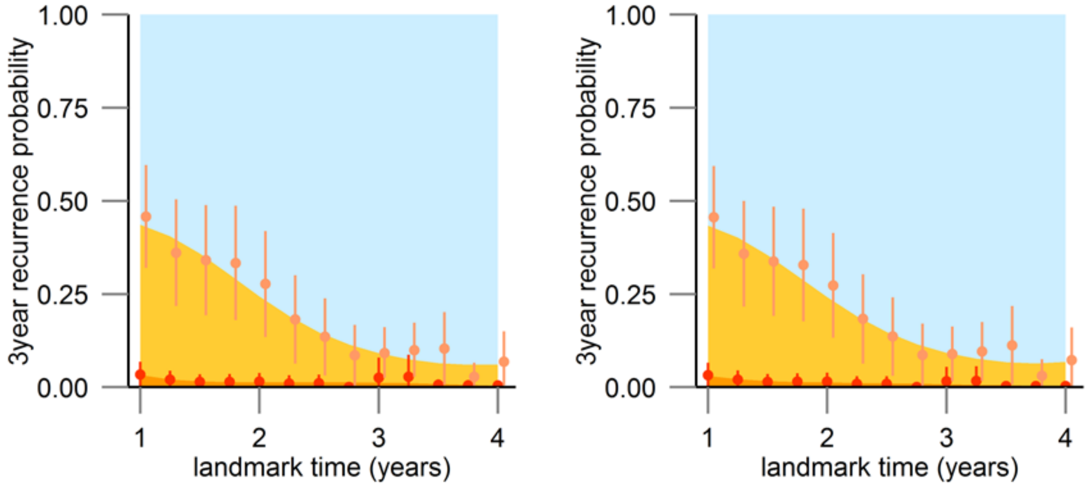

No recurrence before landmark time, single tumor over 2cm

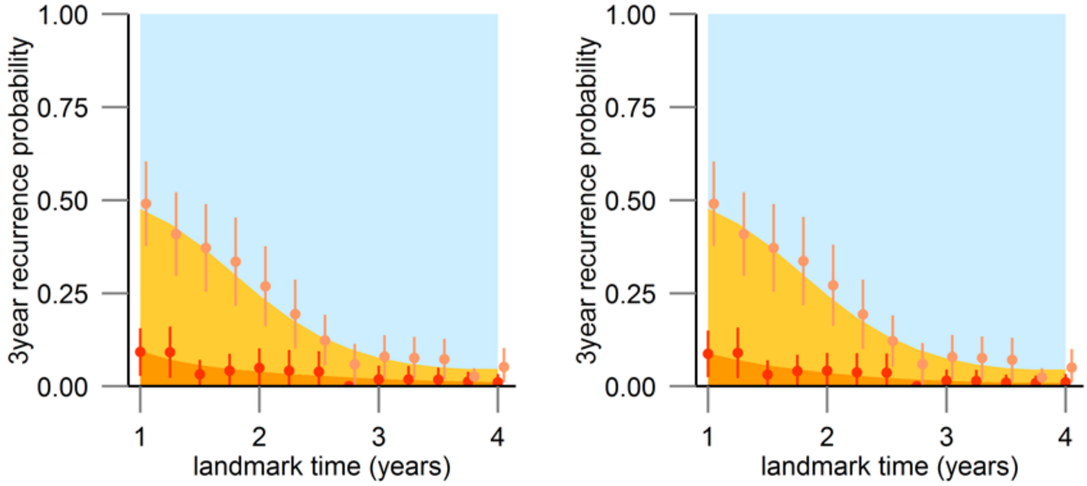

Recurrence occurred before landmark time, single tumor not greater than 2cm

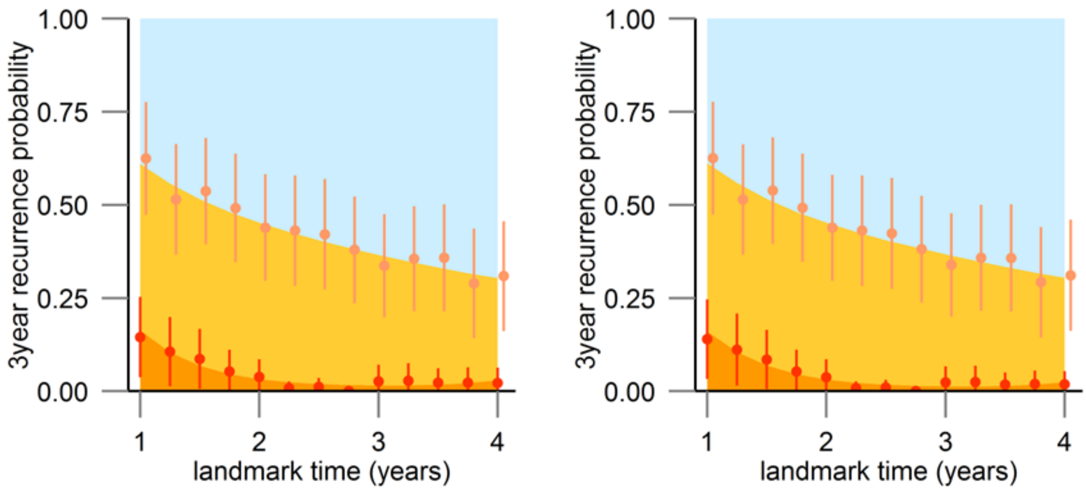

Recurrence occurred before landmark time, single tumor over 2cm

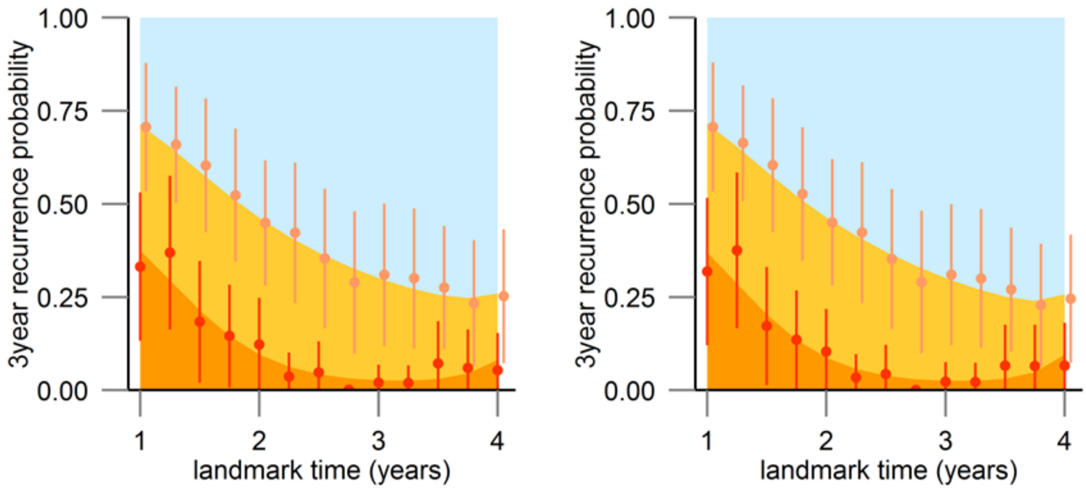

No recurrence before landmark time, multiple tumors not greater than 2cm

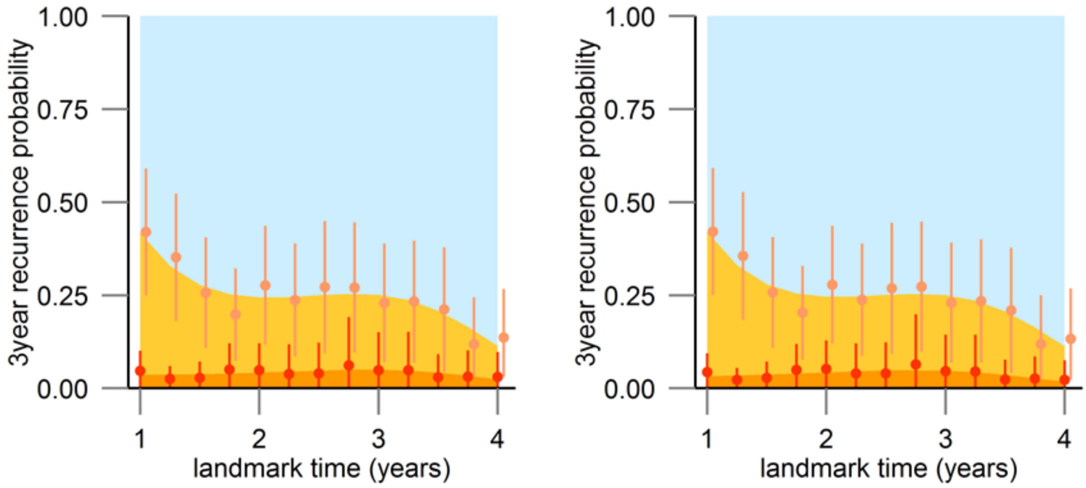

No recurrence before landmark time, multiple tumors over 2cm

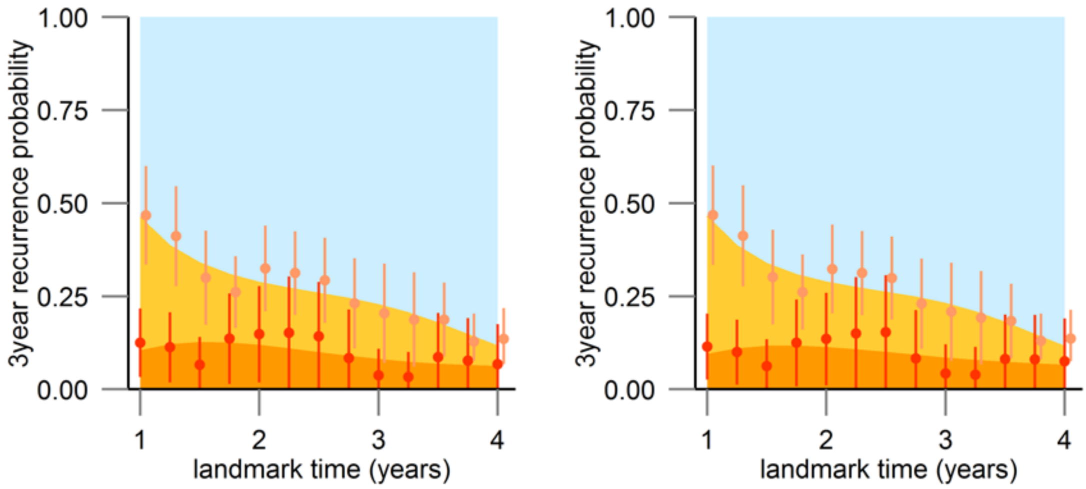

Recurrence occurred before landmark time, multiple tumors not greater than 2cm

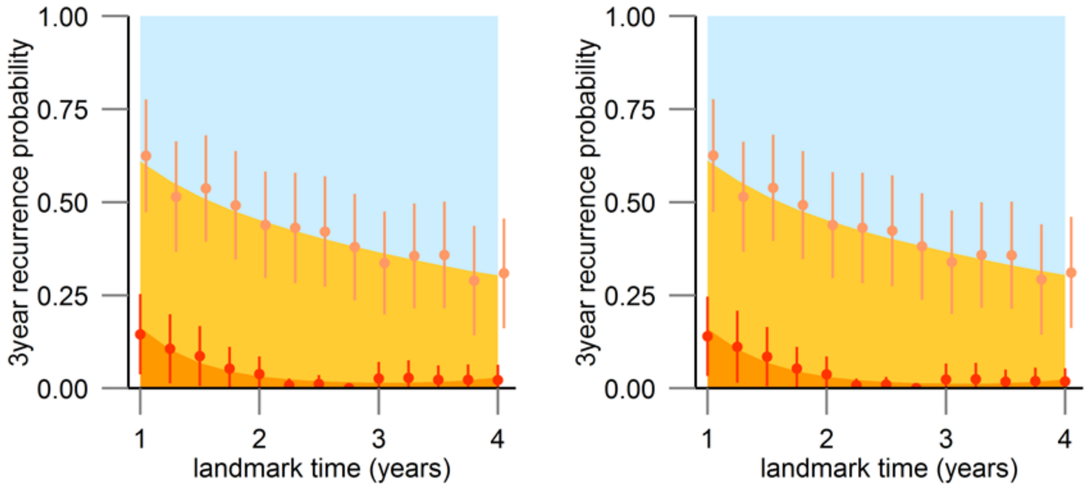

Recurrence occurred before landmark time, multiple tumors over 2cm

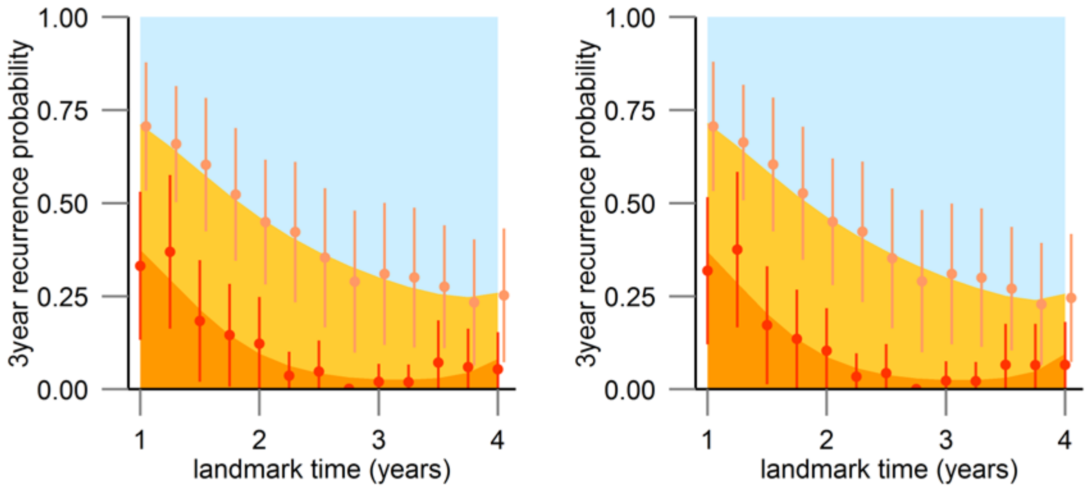

Table S2. Predict 3-year risks of recurrence regarding death as a terminal event.
(a) Parameter estimates in landmarking supermodel using dynamic pseudo observations (DPOs)

	1 time recurrence		2 or more times recurrences		death	
	β	se.	β	se.	β	se.
intercept	-1.43	2.60	-5.14	4.20	-0.78	3.47
time (year)						
s	2.46	4.30	2.82	5.98	-1.84	5.10
s^2	-1.79	2.21	-1.38	2.55	0.79	2.17
s^3	0.25	0.33	0.16	0.32	-0.11	0.28
the number of recurrences (1 or more / 0)						
intercept	1.77	2.68	4.80	4.50	2.60	2.90
s	-3.06	4.19	-3.72	6.48	-1.52	4.25
s^2	1.84	2.05	0.90	2.71	0.60	1.83
s^3	-0.25	0.3	-0.03	0.34	-0.09	0.24
multiple tumors / single tumor						
intercept	3.71	2.33	0.04	4.65	2.34	2.60
s	-7.06	3.59	0.28	6.99	-4.26	3.99
s^2	3.70	1.67	0.27	2.96	1.96	1.83
s^3	-0.53	0.23	-0.05	0.37	-0.24	0.25
the length of tumor (>2cm / ≤2cm)						
intercept	-0.5	2.17	1.29	4.08	-0.33	3.00
s	0.83	3.31	-0.37	6.12	2.65	4.39
s^2	-0.47	1.52	-0.08	2.62	-1.63	1.89
s^3	0.07	0.21	0.04	0.33	0.25	0.25

β: parameter estimates; se.: robust standard error; s:smoothers in the super model.

(b) Predicted 3-year risks of recurrence and death

Stacked graphs are drawn the predicted risks of no recurrence for 3years after landmark time (blue), 1 time recurrence (yellow), 2 or more times recurrence (orange) and death (purple). Circle and error bars show point estimates and 95% CI, respectively, by fixed landmark regression model. Filled areas show point estimates by supermodel.

No recurrence before landmark time, single tumor

Tumor not greater than 2cm | **Tumor over 2cm**

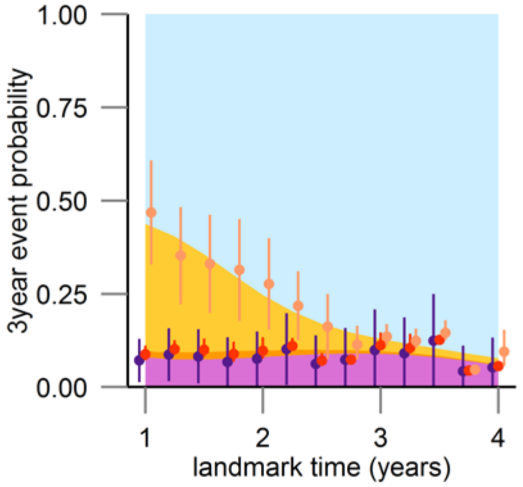

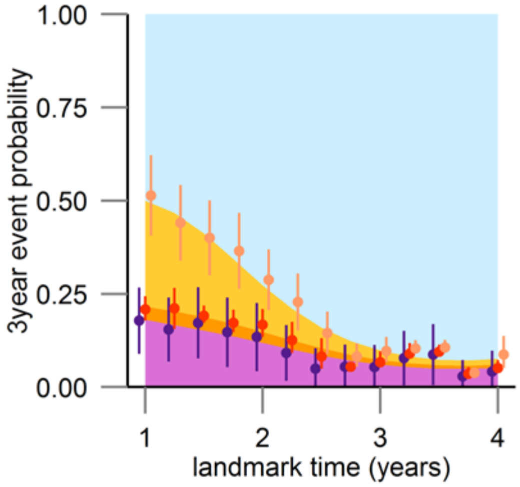

No recurrence before landmark time, multiple tumors

Tumors not greater than 2cm | **Tumors over 2cm**

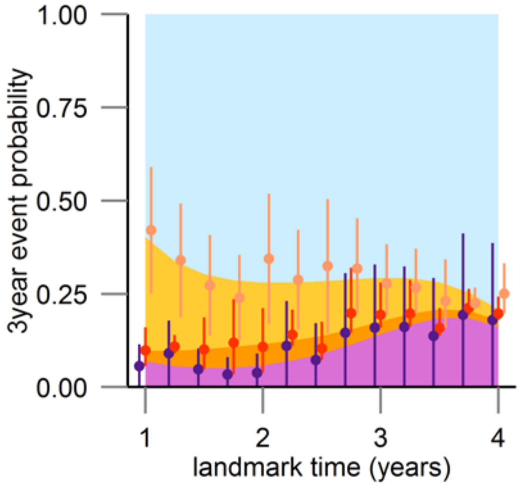

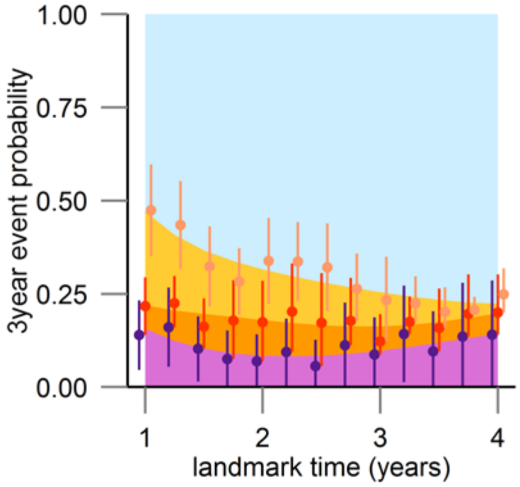

Recurrence occurred before landmark time, single tumor

Tumor not greater than 2cm

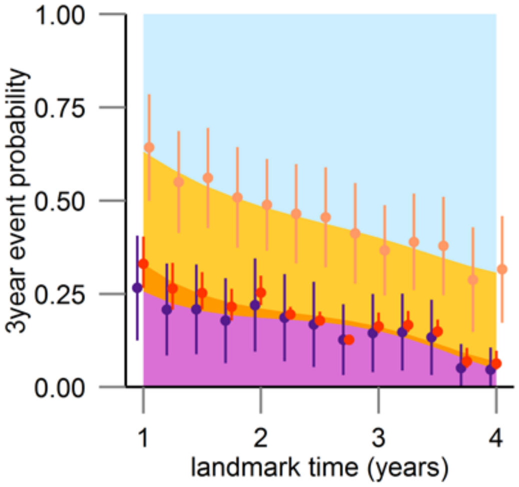

Tumor over 2cm

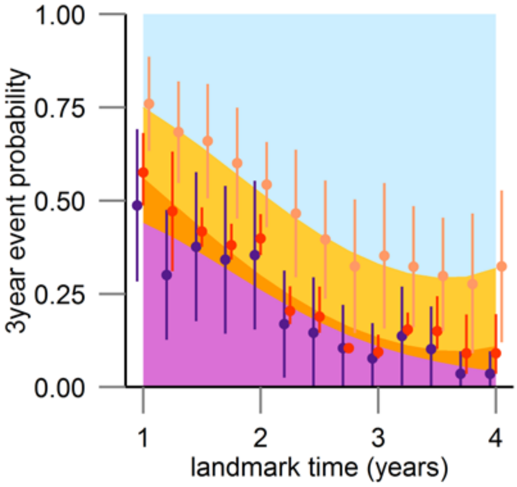

Recurrence occurred before landmark time, multiple tumors

Tumors not greater than 2cm

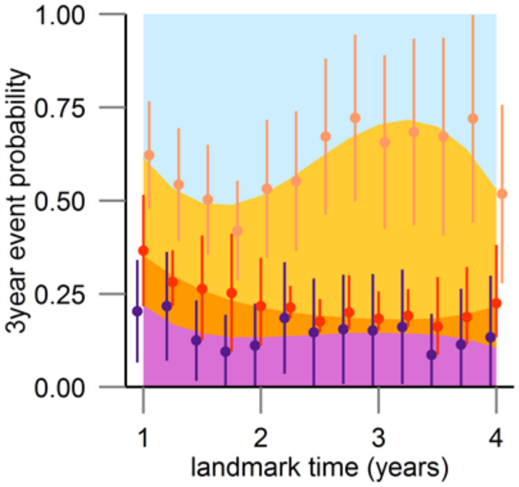

Tumors over 2cm

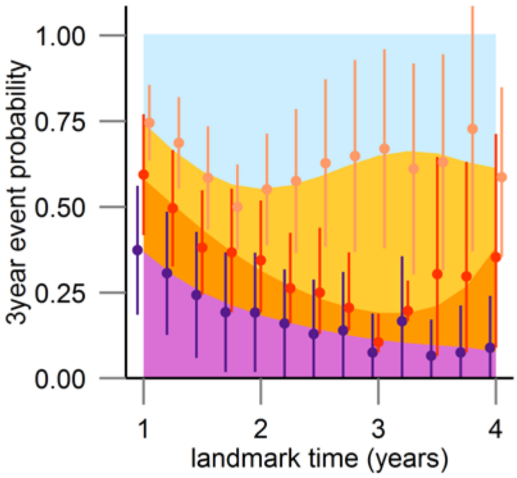

Supplement: Supplementary file 2 — Supplemental tables and figures of proposed DPOs to a real example. (PDF 885 kb) [file 12874_2019_677_MOESM2_ESM.pdf]
